# Parylene C-Based, Breathable Tattoo Electrodes for High-Quality Bio-Potential Measurements

**DOI:** 10.3389/fbioe.2022.820217

**Published:** 2022-03-23

**Authors:** Andrea Spanu, Antonello Mascia, Giulia Baldazzi, Benji Fenech-Salerno, Felice Torrisi, Graziana Viola, Annalisa Bonfiglio, Piero Cosseddu, Danilo Pani

**Affiliations:** ^1^ Department of Electrical and Electronic Engineering, University of Cagliari, Cagliari, Italy; ^2^ Department of Informatics, Bioengineering, Robotics and Systems Engineering Genova, University of Genova, Cagliari, Italy; ^3^ Department of Chemistry, Molecular Sciences Research Hub, Imperial College London, London, United Kingdom; ^4^ Division of Cardiology, San Francesco Hospital, Nuoro, Italy

**Keywords:** breathable electrodes, tattoo electronics, bio-potential, electrocardiography, Parylene C

## Abstract

A breathable tattoo electrode for bio-potential recording based on a Parylene C nanofilm is presented in this study. The proposed approach allows for the fabrication of micro-perforated epidermal submicrometer-thick electrodes that conjugate the unobtrusiveness of Parylene C nanofilms and the very important feature of breathability. The electrodes were fully validated for electrocardiography (ECG) measurements showing performance comparable to that of conventional disposable gelled Ag/AgCl electrodes, with no visible negative effect on the skin even many hours after their application. This result introduces interesting perspectives in the field of epidermal electronics, particularly in applications where critical on-body measurements are involved.

## Introduction

Epidermal electronic, or “tattoo electronic”, is undoubtedly one of the most interesting technological approaches conceived in the field of wearable electronics over the past 10 years. Its introduction dates back to the seminal paper of Kim et al. (Kim et al., 2011), a complex work where an electronic system was proposed and specifically engineered in order to host several devices and sensors, such as LEDs, temperature sensors and strain gauges, operated in direct contact with the skin. Several approaches have been proposed to target different biomedical applications from bio-sensing and chemical sensing (Jia e al., 2013; Bandodkar et al., 2014; Imani et al., 2016; Lee et al., 2017; Alberto et al., 2020), to pressure sensing (Lee et al., 2016), and clinical applications such as the monitoring of wound healing (Hattori et al., 2014). However, the most studied healthcare application where tattoo sensors showed the most significant impact is the recording of bio-potentials from the surface of the skin, such as surface electromyography, electroencephalography, electrooculography and electrocardiography (ECG) (Jeong et al., 2013; Bareket et al., 2016; Casson et al., 2017; Guo et al., 2017; Inzelberg et al., 2018; Shustak et al., 2019). In fact, in those specific applications the conformal contact with the skin (without the use of any conductive gel) and the feature of being unobtrusive are particularly convenient and appealing. In particular, the main requirements of a tattoo system for bio-monitoring applications are: bio-compatibility and chemical inertness (to minimise adverse skin responses), skin conformability (to enhance effective electrode/skin interface adhesion thanks to the maximization of the contact surface, thus greatly improving signal acquisition and minimising motion artefacts, as theoretically demonstrated by (Wang et al., 2017; Wang et al., 2018), and breathability, especially for long-term monitoring, such as in an intensive care unit or dynamic ECG (Holter) applications and in critical applications such as neonatal care and monitoring of heavily wounded or burnt patients. Some of the recently proposed approaches are characterised by low-cost materials and fairly simple fabrication techniques (Zucca et al., 2015; Ferrari et al., 2018; Ferrari et al., 2020; Taccola et al., 2021); others are ultra-thin and highly conformable (Ameri et al., 2017; Ameri et al., 2018; Guo et al., 2019; Ha et al., 2019), highly stretchable thanks to the integration of metal electrodes in elastomers (Shahandashti et al., 2019), or characterised by high breathability because of the use of innovative materials and relatively complex fabrication methods (Miyamoto et al., 2017; Fan et al., 2018; Ferrari et al., 2018; Jiang et al., 2019; Liu et al., 2019). An interesting approach that is worth mentioning is indeed represented by “serpentine electrodes” (Jang et al., 2014; Chae et al., 2019; Wang et al., 2020), which allow to greatly improve the breathability in structures where non-breathable materials are used. However, a definitive solution that is able to provide at the same time easy-to-fabricate, breathable and ultra-conformable dry electrodes for the detection of bio-potentials from the surface of the skin has not been proposed yet. The goal of our approach is to identify a solution to these issues using one of the most promising materials in the biomedical field, i.e. Parylene C. In fact, Parylene C has been successfully employed for several biomedical applications, such as cellular interfacing (Spanu et al., 2021a) and the realisation of both conformable electronic devices and electrodes in direct contact with the skin because of its bio-compatibility, chemical inertness and the possibility of obtaining ultra-thin sub-micrometre layers through a reliable and high-throughput chemical vapour deposition technique (Nawrocki e al., 2016; Peng et al., 2016; Nawrocki e al., 2018; Viola et al., 2018). Despite very good chemical and mechanical properties, Parylene C lacks breathability. In fact, this material is routinely used as a protection layer in many electronic applications thanks to its very good chemical robustness, which makes it an ideal barrier against water and oxygen interdiffusion. To overcome this limitation, while retaining the features of the Parylene C nanofilms, we propose an easy, highly reproducible large area perforation technique that can be used to obtain ultra-conformable, sub-micrometre electrodes that are unobtrusive and breathable, without an adhesive or conductive hydrogel. Using this technique, we designed and validated breathable tattoo electrodes for ECG signal detection based on biocompatible materials (i.e., Parylene C and Ag), with the goal of obtaining the high-quality recordings required for a proper clinical ECG interpretation and diagnosis, and at the same time helping to minimize the typical skin irritation effects caused by conductive gel and glue in standard electrodes. On this basis, the performance of breathable tattoo electrodes in this work was compared with those of both non-breathable dry Parylene C tattoo electrodes and commercial disposable gelled Ag/AgCl electrodes in terms of permeability, skin-electrode impedance and ECG recording, revealing excellent results.

## Materials and Methods

### Electrode Fabrication

**FIGURE 1:**
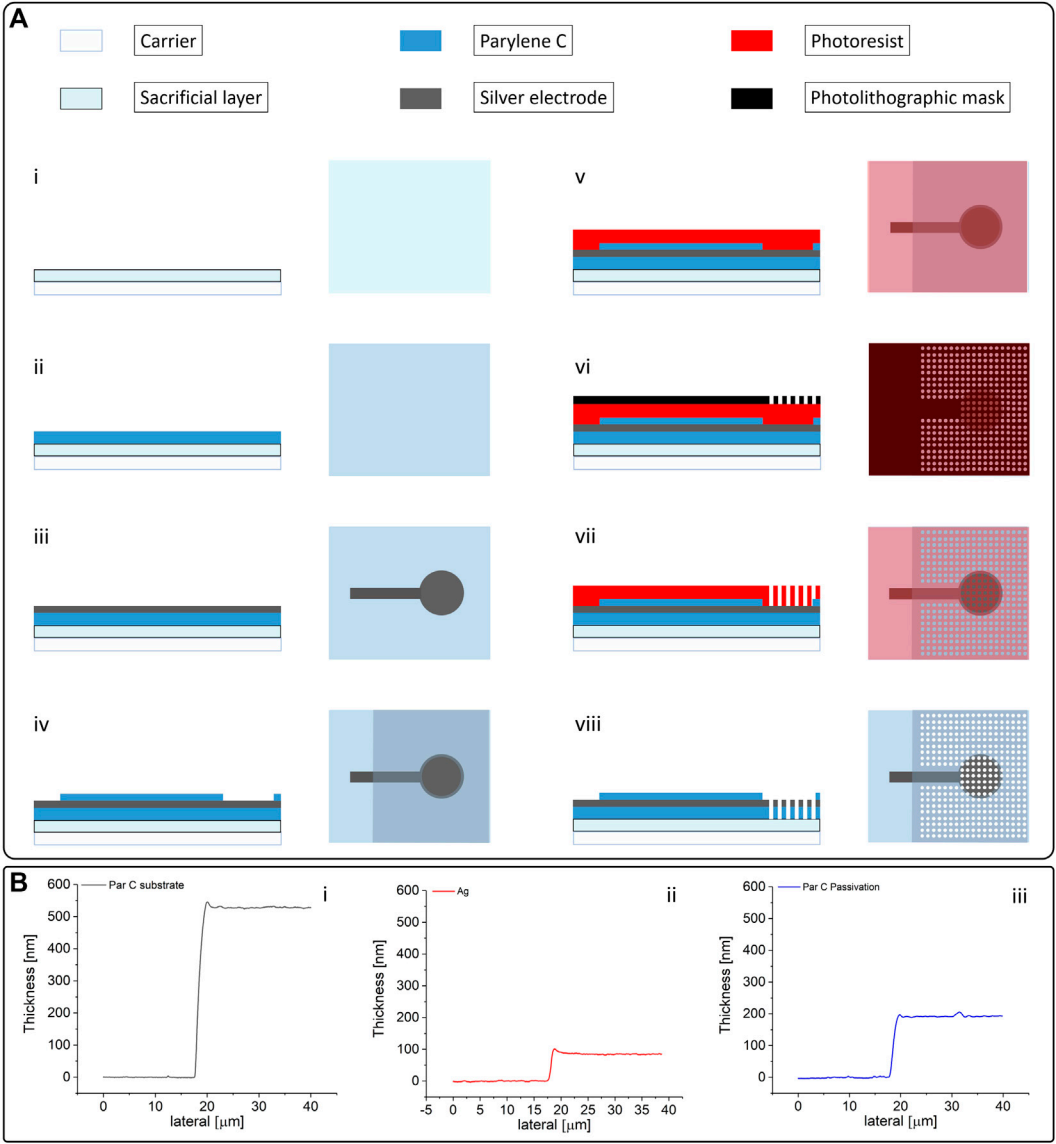
**(A)** Fabrication process of the breathable Parylene C-based electrodes (with materials). The fabrication starts with the deposition of a PVA sacrificial layer onto the PET carrier (i); the Parylene C that acts as the substrate is deposited on the sacrificial layer through chemical vapor deposition (ii); deposition and patterning of the Ag electrode (iii); another layer of Parylene C, which acts as a passivation layer, is then deposited on the electrode with the exception of the connector and the recording regions (iv); the perforation is performed using a photolithographically patterned photoresist layer as a mask (v, vi,vii) to selectively remove Ag and Parylene C using a combination of wet etching (for the Ag) and plasma oxygen etching (for the Parylene C); after the perforation is complete, the photoresist is removed (viii) and the electrode is ready to be peeled-off and transferred to the paper. **(B)** Thickness of the three layers constituting the electrodes, namely the first Parylene C layer that acts as the substrate (i), the Ag layer (ii), and the Parylene C passivation layer (iii).

All electrodes were fabricated on a 250 µm thick polyethylene terephthalate (PET) carrier. At first, a sacrificial layer of poly (vinyl alcohol) (PVA; a 6 wt% in a PVA solution in deionized water) was spin-coated on the substrate and baked for 5 min at 90°C. This PVA layer is needed to perform all of the fabrication steps without premature detachment of the nanofilm. A first layer of Parylene C of approximately 500 nm was subsequently deposited onto the carrier. The negative pattern of the electrode array was then obtained using a standard photolithographic process. This patterned photoresist film is then covered with 70 nm of evaporated silver (after a slight plasma activation of the surface–60 s at 100 W) and eventually stripped in a sonicated acetone bath. All the electrodes employed in this work had a circular recording area (diameter: 1 cm). After this lift-off process, a second Parylene C layer of approximately 200 nm, which acts as a passivation layer, was deposited on the substrate, with the only exception of the connector of the array and the electrode recording area (which were covered with a polydimethylsiloxane patch during the deposition process). The tattoo patch was then ready for the perforation process. A layer of photoresist was spin-coated on the substrate, patterned with the desired holes density and, after the removal of the silver from within the holes with a quick wet etching using a KI solution, exposed to oxygen plasma (7 min at 200 W). The complete fabrication process is shown in Figure 1A. To evaluate the breathability of the obtained electrodes, two different holes’ diameters, 100 and 50 μm, with a hole density of four holes/mm^2^, were tested. The presented ECG measurements were performed with the patches with the 100 µm-diameter holes.

**FIGURE 2:**
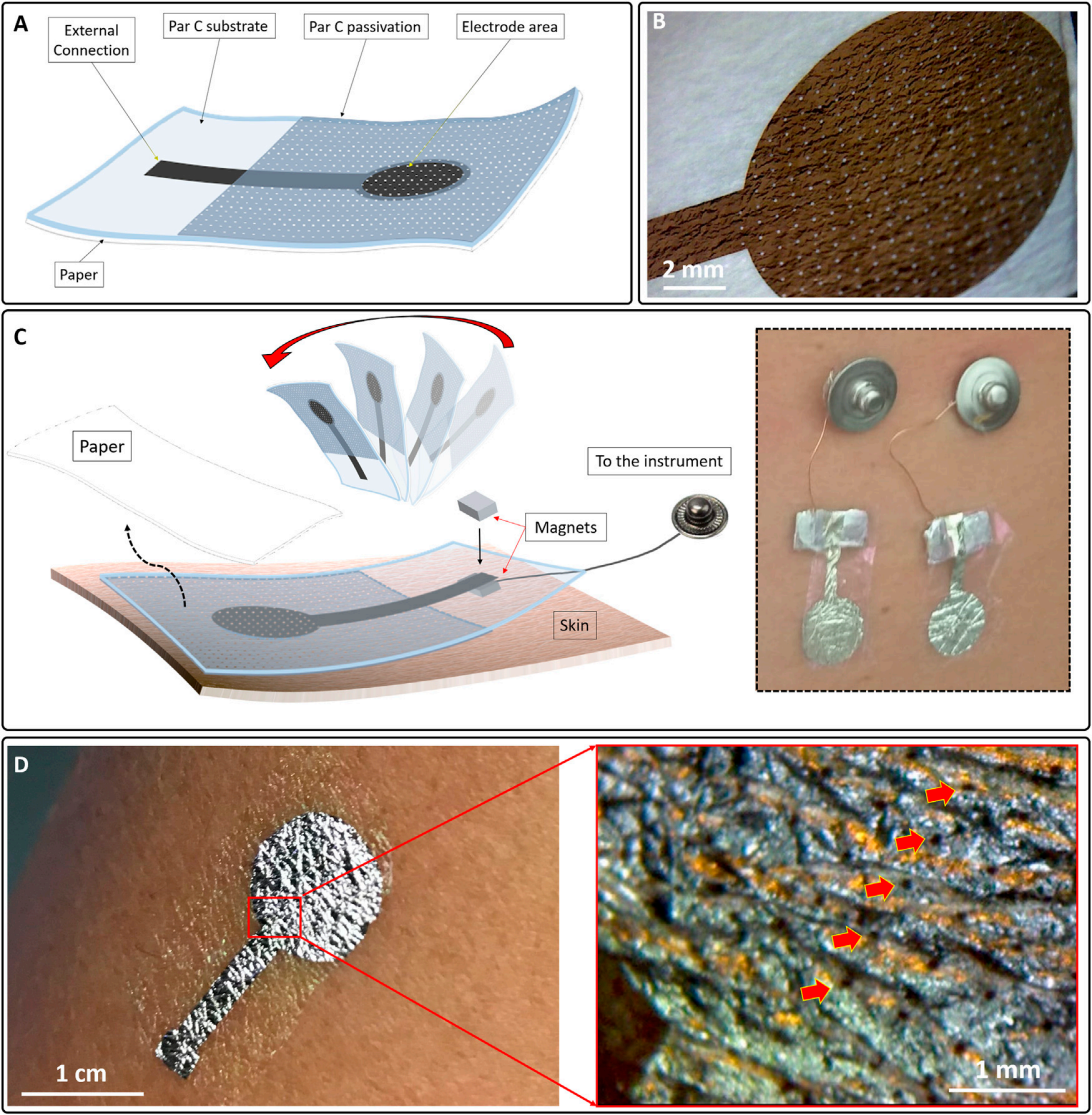
**(A)** Structure of the breathable tattoo electrode after its placing on a piece of paper so that it could be easily transferred to the skin. **(B)** Micrograph of a breathable electrode on paper. The pores are clearly visible. **(C)** Positioning on the skin. The electrode was placed face down on the skin and the paper was removed using a few droplets of deionized water and sliding it away. The electrode contact was placed on a metalized magnet connected to a clip contact (Cinset). A second magnet was placed on top of the first to improve the contact between the film and the first magnet. **(D)** A breathable nano-electrode after its lamination on the skin. (Dinset) Micrograph of the electrode on the skin. Thanks to the sub-micrometer thickness, a conformal interface can be obtained; it is also possible to spot the pores (red arrows).

The final electrode has an overall thickness of approximately 700–800 nm. The thicknesses of the different layers were evaluated on sacrificial substrates using a contact profilometer (Bruker DektakXT), as shown in Figure 1B. The patch was then peeled-off and eventually transferred to a piece of paper (Figures 2A,B) using a small amount of deionised water (this step also promotes the dissolution of the PVA sacrificial layer). After the sample was dried out, the tattoo electrode was ready to be transferred to the skin by simply placing the patch on the desired location and wetting the back of the paper, as depicted in Figure 2C. The paper can then be removed by sliding it away, leaving the patch tightly adhered to the skin. The connection to the recording device is ensured by placing the exposed back contact of the electrode on a small silver-coated neodymium magnet (×3 5 mm with a thickness of 1 mm) connected to a clip contact through a passivated copper wire (Figure 2C inset). Another magnet can be placed on the film to keep it firmly in place. After the recording session, the tattoo can be easily removed from the skin with a piece of wet paper. Figure 2D show a breathable nanofilm after its positioning on the skin.

### Permeability Measurement Setup

Air permeability measurements were performed on a TexTest 3,340 MinAir device at 20°C. Prior to the sample measurements, the device gaskets were wiped down with propan-2-ol and calibrated on a 20 cm^2^ standard calibration plate (358 mm/s, ± 3 mm/s), at 200 Pa. Sample measurements were taken using a pressure drop of 100 Pa with a 5 cm^2^ adapter and relative humidity of 37% or 63%.

### Impedance Measurement Setup

The breathable and standard tattoo electrodes were characterised in terms of skin-electrode contact impedance by using the instrument is an Agilent 4284A precision LCR meter (Agilent Technologies Inc., Santa Clara, CA, United States). A low sinusoidal current was injected on the body, performing a 4-probe measurement in the frequency range between 20 and 500 Hz (Pani et al., 2015). The measurement was performed between the experimental electrode and the parallel of five commercial pre-gelled electrodes (BlueSensor N, Ambu A/S, Denmark), applied after local gentle skin abrasion by a preparation cream (NuPrep, Weaver and Co., CO, United States). The same measurement approach has been used for the assessment of the variation of the impedance over time. In this case, a medical grade impedance-meter with a fixed frequency of 10 Hz (EIM-105 Prep-Check, General Devices) has been employed due to the different placement of the electrodes.

### ECG Signal Acquisition and Experimental Setup

In this study, 18 ECG signals were recorded from four healthy volunteers (age: 32 ± 10, BMI: 22.8 ± 1.9, heart rate: 63 ± 7 bpm). The experimental protocol was conducted following the principles outlined in the Helsinki Declaration of 1975, as revised in 2000. All of the participants gave their written informed consent. Signal acquisition was performed using a 32-channel Porti7 electrophysiological recording system (TMSI, The Netherlands) at a sampling rate of 2048 Hz, with an effective bandwidth of 553 Hz. To accurately assess the cardiac rhythm, lead II was chosen for all ECG signal acquisitions as it is commonly used to record the rhythm strip and it provides good P wave representation (Meek and Morris, 2002a). Specifically, lead II was recorded by adopting a pair of electrodes on the torso according to Holter electrode placement configuration, as previously demonstrated (Pani et al., 2015). The LL electrode was placed on the left anterior axillary line, while the RA electrode was placed slightly under the right manubrium. The ground electrode was placed near the right hip. The different pairs were interleaved by preserving the same inter-electrode distance to simultaneously record the cardiac electrical activity by three different pairs of electrodes (breathable tattoo, non-breathable tattoo and commercial gelled Ag/AgCl) with limited impact on signal morphology and amplitude.

### ECG Signal Quality Evaluation and Processing

Comparative analyses were performed on 15-s long ECG traces, consisting of three simultaneous recordings from the three electrode pairs. The quality of the recorded ECG signals was assessed by exploiting the quantitative figures of merit and from a clinical perspective. To this aim, some signal processing steps were implemented.

**FIGURE 3:**
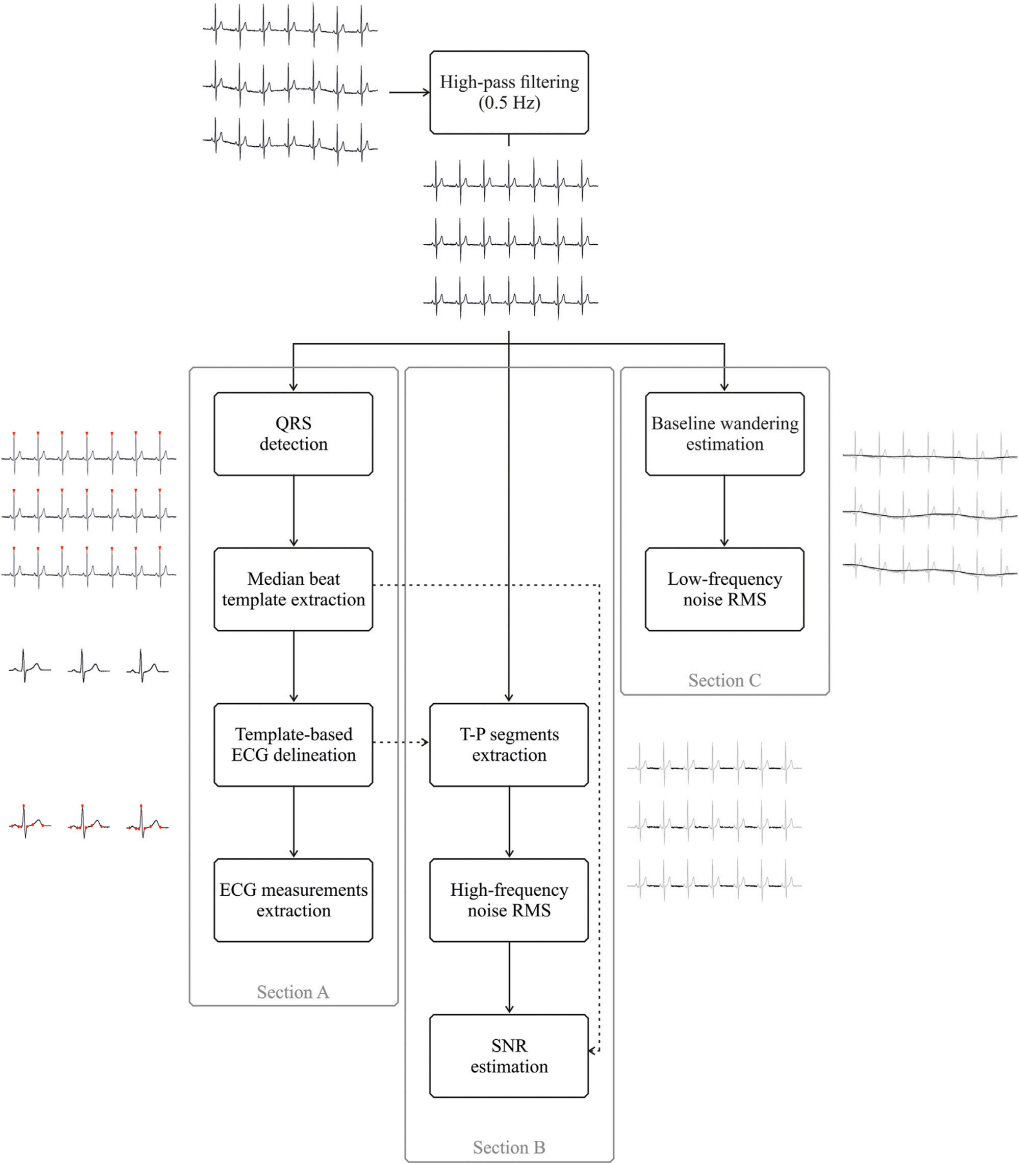
Schematic representation of the different signal processing steps adopted for ECG measurement extraction (Section A) and for signal-to-noise ratio (SNR), high-frequency and low-frequency noise root-mean square estimation (Sections B and C, respectively). All sections share the same initial processing step, i.e., high-pass filtering at 0.5 Hz, despite the different purposes. Furthermore, the dashed arrow pointing from the template-based ECG delineation (Section A) to T−P segments extraction (Section B) indicates that T−P intervals were identified in high-pass filtered ECGs by exploiting T and P wave delineation on median beat templates, whereas the dashed arrow pointing from the median beat template extraction (Section A) to SNR estimation (Section B) refers to the adoption of the peak-to-peak amplitude of the median beat template for the SNR computation.

First, the baseline wander artefact was removed using a 2nd order IIR Butterworth high-pass filter with a cut-off frequency of 0.5 Hz, which has been proven to be effective for this purpose, especially for medical applications (Lenis et al., 2017), despite being more conservative than standard settings (i.e. 0.67 Hz) (Kligfield et al., 2007). This filtering stage was performed both in the forward and reverse directions, thus providing a digital filter with zero phase distortion. Furthermore, considering the goal to compare the performance of the different electrodes, no other filtering step, such as notch filters to suppress powerline interference, was introduced as the amount of powerline noise is related to the skin-contact electrode impedance and the stability of such an interface. A state-of-the-art wavelet-based QRS detector (Martínez, et al., 2004) was then applied and all highly correlated beats (Pearson’s correlation coefficient greater than 0.95) were identified and used to obtain a median beat template by synchronized averaging. Median beat analysis reduces the random noise hampering the robust measurement of the main waveform-based features (Johannesen et al., 2013), as typical in computer-aided diagnosis software tools. Based on aligned R peaks locations, an ECG synthetic trace was created for each electrode type by repeating the median beat in every QRS location to further delineate the main ECG waveforms related to each template using the previously developed wavelet-based ECG delineator (Martínez, et al., 2004), which cannot be directly used for the delineation of a single median beat. Figure 3 (Section A) depicts the main processing steps needed for the median template generation and delineation.

Similarly to (Castañ o et al., 2019), on the median beat template of each trace three kinds of ECG measurements were considered on the median beat template of each trace: 1) the peak-to-peak amplitude of the QRS complex; 2) the duration of each main ECG waveform (P wave, T wave and QRS complex); 3) all possible intervals that could be useful in a clinical setting, such as the PQ interval, R–R interval and QTc interval, the latter corrected using the Bazett’s formula (Bazett, 1920) to be insensitive to heart rates. Specifically, the P wave is the first deflection from the isoelectric baseline in each cardiac cycle, which represents atrial depolarization, and it is followed by a sharp sequence of waves, i.e. the QRS complex and the T wave, embodying ventricular depolarization and repolarization, respectively. For healthy subjects, P wave and QRS complex durations are typically less than 120 and 100 m, respectively (S. Meek, F. Morris and Morris, 2002b; Goldberg et al., 2017). The PQ interval reflects the atrio–ventricular (AV) conduction and, which can be related to AV delay or first-degree AV block in the case of prolongation or as an accessory pathway in the case of shortening. Normal PQ intervals range from 120 to 200 m (S. Meek, F. Morris, 2002b; Goldberg et al., 2017). The QT interval represents the time of ventricular depolarization and repolarization and its alteration could reflect inherited disease such as long or short QT syndromes. In normal conditions, QT intervals are between 350 and 450 m (S. Meek. Morris, 2002b), whereas QTc ranges from 330 to 440 m (Goldberg et al., 2017). Alternatively, R–R intervals represent the core of the heart rate variability studies and sinus arrhythmias investigations. Assuming a normal heart rate between 60 and 100 bpm (Goldberg et al., 2017), R–R intervals can span approximately between 600 and 1,000 m. The selection of the median beat, compared to an ensemble average (Castañ o et al., 2019), improves the robustness against outliers and is commonly adopted in the commercial software for ECG automated analysis, as the GE Healthcare Marquette 12SL ECG Analysis Program (GE Healthcare, Wawatosa, WI, United States), e.g., in (Pani et al., 2016), and it is also contemplated by ECG communication standards (Rubel et al., 2021).

Other signal processing steps were implemented to quantify the signal-to-noise ratio (SNR), the low-frequency and high-frequency noise affecting the different ECG recordings. The low-frequency noise entity was estimated by considering the root-mean-square (RMS) value of the baseline wandering, which was obtained by subtracting the high-pass filtered signal from the corresponding raw recording, thus implementing a low-pass filtering stage with the same cut-off frequency of 0.5 Hz. Specifically, RMS was approximated by the standard deviation of the 15s-long baseline wander artefact after its centering (i.e. the removal of the signal offset). Alternatively, high-frequency noise content was assessed by discarding all ECG physiological deflections on the high-pass filtered recordings and considering the RMS on the isoelectric intervals. In this case, RMS was estimated on each isoelectric interval, which was identified as the signal portion between the end of the T wave, and the onset of the consecutive P wave on the delineated ECG and the median RMS value of the different intervals was considered for each ECG recording as high-frequency noise measure. Furthermore, being the SNR typically adopted for the assessment of ECG signal quality (Castaño et al., 2019), it was also included in this study. Specifically, SNR was computed following standard definitions, as

SNR[dB]=20⁡ log10(App4σ)

where A_pp_ identifies the ECG signal contribution for each trace as the peak-to-peak amplitude of the median beat template, whereas σ is related to the high-frequency noise content, and as such it was evaluated as the median RMS derived from the isoelectric intervals.

Figure 3B reports the processing stages implemented for the high-frequency noise and SNR estimation, whereas Section C shows the steps needed for the low-frequency noise estimation.

Statistical analysis was performed on each index and ECG measurement to identify any discrepancy in the signals acquired by the three electrodes. In this regard, in the case of the normality of the distributions was not verified by the Lilliefors test, the Kruskal–Wallis non-parametric test was adopted to reveal any difference in the group, otherwise the one-way ANOVA was used. Similarly, when a statistical difference was observed in the group, pairwise comparisons were conducted by the non-parametric Wilcoxon signed rank test or the paired-sample Student’s t-test, according to the normality of the distribution. In all statistical analyses, a significance level of 5% was considered.

Furthermore, to provide preliminary data on the applicability of the developed electrode technology for long-lasting recordings, low-frequency and high-frequency noise levels were estimated on the ECG signals acquired on a single subject over 9 hours.

All processing and statistical analysis was performed using Matlab 2018b (The Mathworks, MA, United States).

### Clinical Evaluation of the ECG Recordings

To assess the quality of the recordings obtained by the three different electrode technologies, an expert cardiologist visually inspected all 15 s-long high-pass filtered ECG traces and the resulting median beat template, providing a score between one and ten. The score was assigned according to the noise level, the intelligibility of the signals and the morphology of the different waveforms of the ECGs. Remarkably, no screen filters (such as low-pass or notch filters) were introduced before the visual inspection. Moreover, the cardiologist was asked to verify if any clinically evident and relevant difference between the ECG measurements automatically extracted on the delineated ECG recorded with the different electrodes was present.

## Results

### Permeability Measurements

**FIGURE 4:**
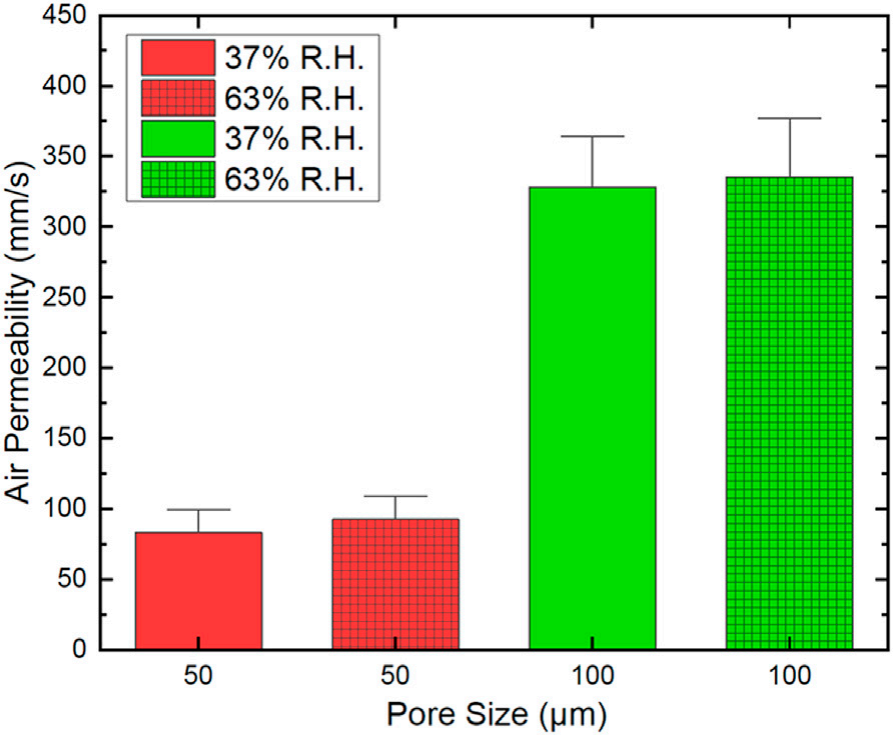
Air permeability of two sets of breathable Parylene C nanofilms with different pore sizes evaluated for two values of relative humidity.

Air permeability measurements taken at 100 Pa and 37% relative humidity showed mean air permeability (δ) of 99 mm/s and 364 mm/s for 50 and 100 µm pores, respectively. The δ increased respectively to 109 mm/s and 377 mm/s at 63% relative humidity, as shown in Figure 4. Previous work on textiles suggests that some materials may swell with an increase in relative humidity, which in turn affects the porosity of the material (Gibson et al., 1999). This may be responsible for the increase in air permeability at higher relative humidity in our samples. The air permeability of these breathable nanofilms exceeds that of a range of novel wearable structures, including electronic skin (120 mm/s at ≥125 Pa) (Peng et al., 2020), paper electrodes (330 mm/s at 300 Pa) (Yang and Lu, 2019), novel wound dressings (324 mm/s at 200 Pa) (Liu et al., 2018) and electronic textiles (88–160 mm/s at 100 Pa) (Cao et al., 2018; Qiang et al., 2019; Wang et al., 2019). Moreover, these values are greater than some common textiles, such as jeans (∼50 mm/s at 300 Pa) (Yang and Lu, 2019), and approaches the values obtained for knitted clothing (>500 mm/s at 100 Pa) (Selli and Turhan, 2017).

### Skin-Electrode Impedance Characterization

**FIGURE 5:**
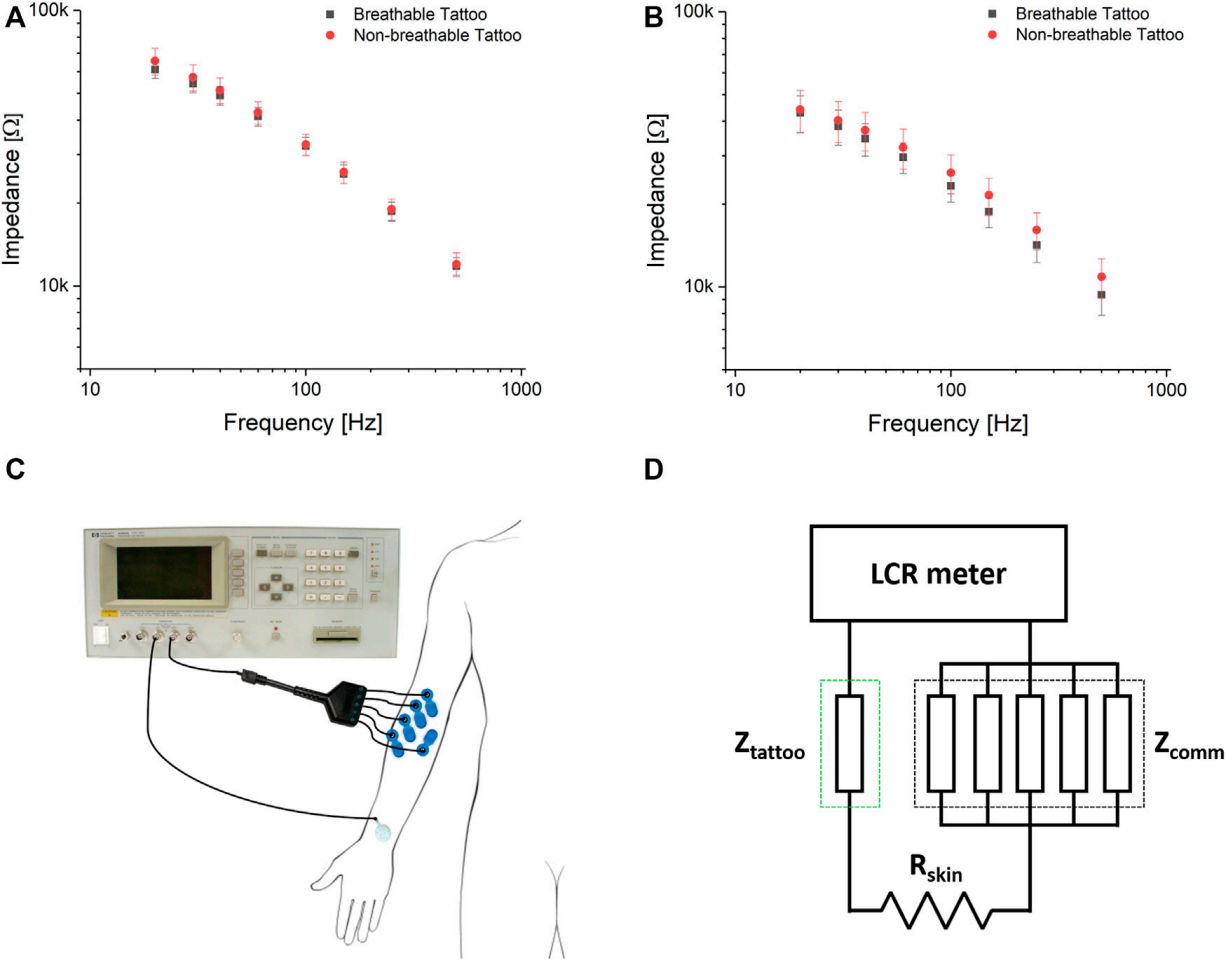
Frequency dependency of the skin-electrode contact impedance measured on the same subject (and on the same spot in the forearm) on two different days **(A,B)**, using 20 electrodes each session. **(C,D)**: positioning of the electrodes for the impedance characterization and electrical scheme, respectively. The Z_tattoo_ has been evaluated against the parallel of five commercial electrodes (Z_comm_) in order to make the latter negligible and thus having a more accurate measurement.

The impedance was measured in two different days (Figures 5A,B), on the forearm of the same subject to minimise the differences due to the inter-person variability of the skin characteristics. For each day, 20 tattoo electrodes were alternately tested by positioning them on the skin using a few droplets of deionized water. As depicted in Figure 5C, the measurement was performed between the experimental electrode and the parallel of five commercial pre-gelled electrodes. In this way, the measured impedance corresponds to the series of: 1) the impedance between the skin and the experimental electrode; 2) the impedance of the body; 3) the impedance between the skin and the parallel of the five commercial pre-gelled electrodes (Figure 5D). However, the body impedance is negligible with respect to that of any skin-electrode interface (Webster, 2010). Moreover, the parallel of five commercial pre-gelled electrodes provides an overall contribution equal to one fifth of the single skin-electrode contact impedance, which is already significantly low for this kind of electrodes after skin preparation. Therefore, the largest part of the measured impedance can be ascribed to the skin-tattoo contact. The skin-electrode contact impedance showed good reproducibility within the same recording session, with marked differences between the two sessions, which was highly predictable considering the strong dependence of the skin impedance from several factors, both physiological and environmental, regardless of the electrode technology. Values in the range of 40–60 kΩ at 20 Hz were recorded. Overall, the contact impedance of the electrodes is comparable (or even lower) with that of other epidermal electrodes that can be found in literature (Ferrari et al., 2018; Bareket et al., 2016. No relevant differences between the breathable and standard tattoo electrodes in terms of impedance were observed.

### Comparative Indexes and ECG Measurements

Table 1 shows the ECG measurements extracted from each median beat in terms of median values and 25th and 75th percentiles over the whole dataset. In this regard, statistical analyses revealed that no significant differences were observed among the ECG measurements computed on the signals recorded by the different electrodes (*p* > 0.05 for all the ECG measurements). Moreover, from a clinical perspective, all ECG intervals were compliant with those expected for healthy subjects and similar, especially for the R–R intervals, suggesting that the adoption of these new electrodes may be applicable to heart rate variability analyses. Finally, all ECG traces and beat templates were interpretable by the cardiologist, i.e. the P and T waves were clearly visible.

**TABLE 1:** ECG measurements for each electrode type in terms of median values and 25th and 75th percentiles (in brackets).

	Non-breathable tattoo	Breathable tattoo	Gelled Ag/AgCl
QRS amplitude (mV)	3.23	3.16	2.96
	[2.96; 3.28]	[2.85; 3.25]	[2.85; 3.21]
P duration (ms)	117.19	119.14	117.19
	[113.28; 125.00]	[113.28; 125.00]	[113.28; 125.00]
QRS duration (ms)	83.98	97.66	85.94
	[66.41; 101.56]	[66.41; 101.56]	[66.41; 101.56]
T duration (ms)	181.64	175.78	189.45
	[171.88; 195.31]	[167.97; 187.50]	[179.69; 199.22]
PQ interval (ms)	158.20	156.25	156.25
	[152.34; 164.06]	[144.53; 164.06]	[152.34; 160.16]
RR interval (ms)	914.06	913.82	912.60
	[885.25; 1,050.78]	[885.25; 1,050.78]	[885.25; 1,050.78]
QTc interval (ms)	388.52	388.38	386.08
	[366.55; 393.76]	[377.15; 396.61]	[365.83; 396.42]

**FIGURE 6:**
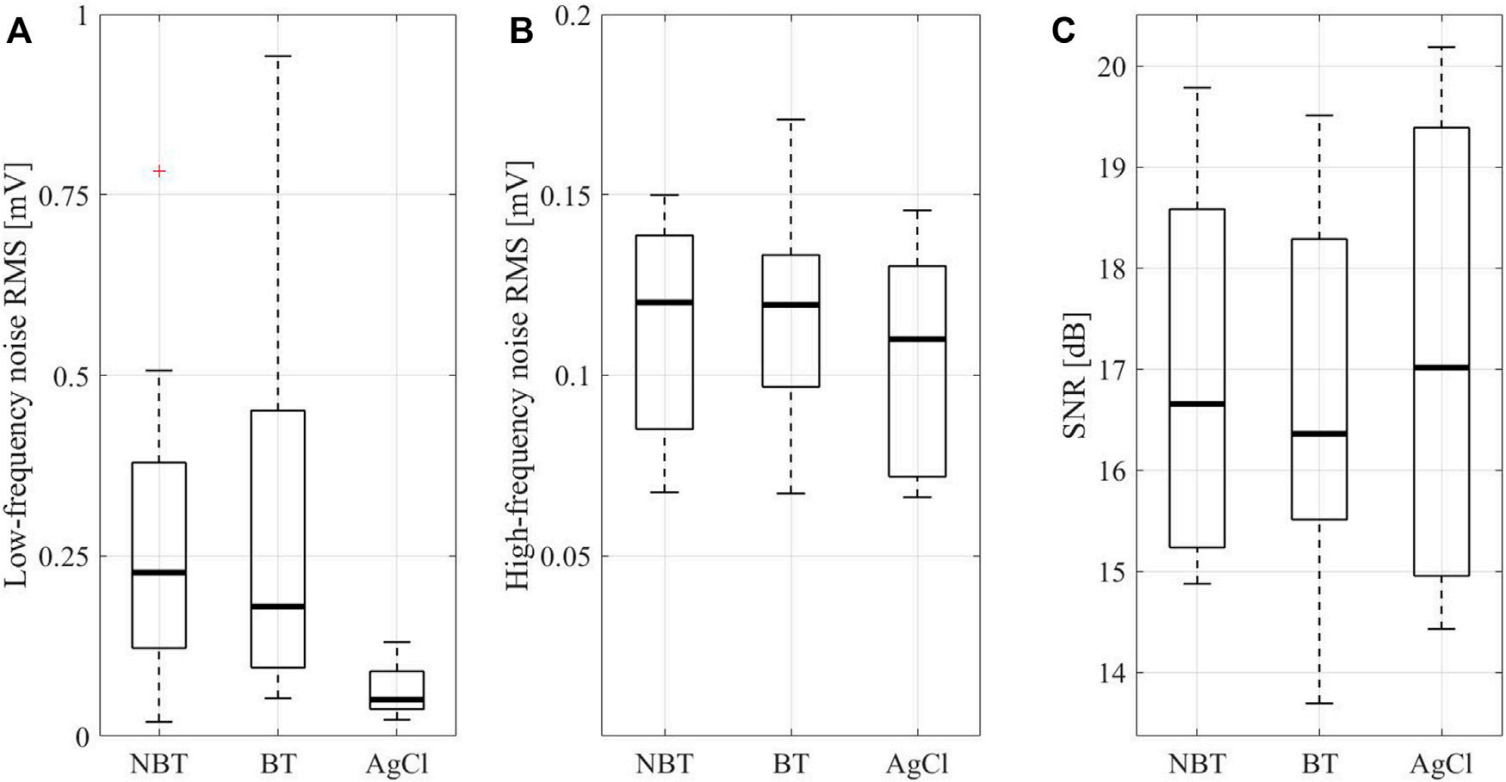
Root-mean-square values for low-frequency (less than 0.5 Hz, **(A)** and high-frequency (greater than 0.5 Hz, **(B)** noises and the SNR values **(C)** characterizing the ECG recordings acquired by non-breathable tattoo (NBT), breathable tattoo (BT), and commercial gelled Ag/AgCl electrodes (AgCl). In the low-frequency noise root-mean-square representation, two outliers for non-breathable tattoo (close to 1.4 and 1.8 mV, respectively) and one for breathable tattoo (near 1.2 mV) were not represented for the sake of clarity.

Figure 6 similarly reports the low-frequency and high-frequency noise RMS along with the SNR estimations on the three different electrode types in terms of boxplots, including median values (central thick line), 25th and the 75th percentiles (lower and upper box edges, respectively), extreme values for each distribution (whiskers) and outliers (red crosses). With regard to the baseline wander artefact (Figure 6A), statistical analysis showed a significant difference among acquisitions performed by breathable tattoo, non-breathable tattoo and disposable gelled Ag/AgCl electrodes (*p* < 0.0000). Specifically, breathable and non-breathable tattoo electrodes showed significantly greater RMS values than gelled electrodes (*p* < 0.0005), assuming similar values to each other (*p* > 0.05).

**FIGURE 7:**
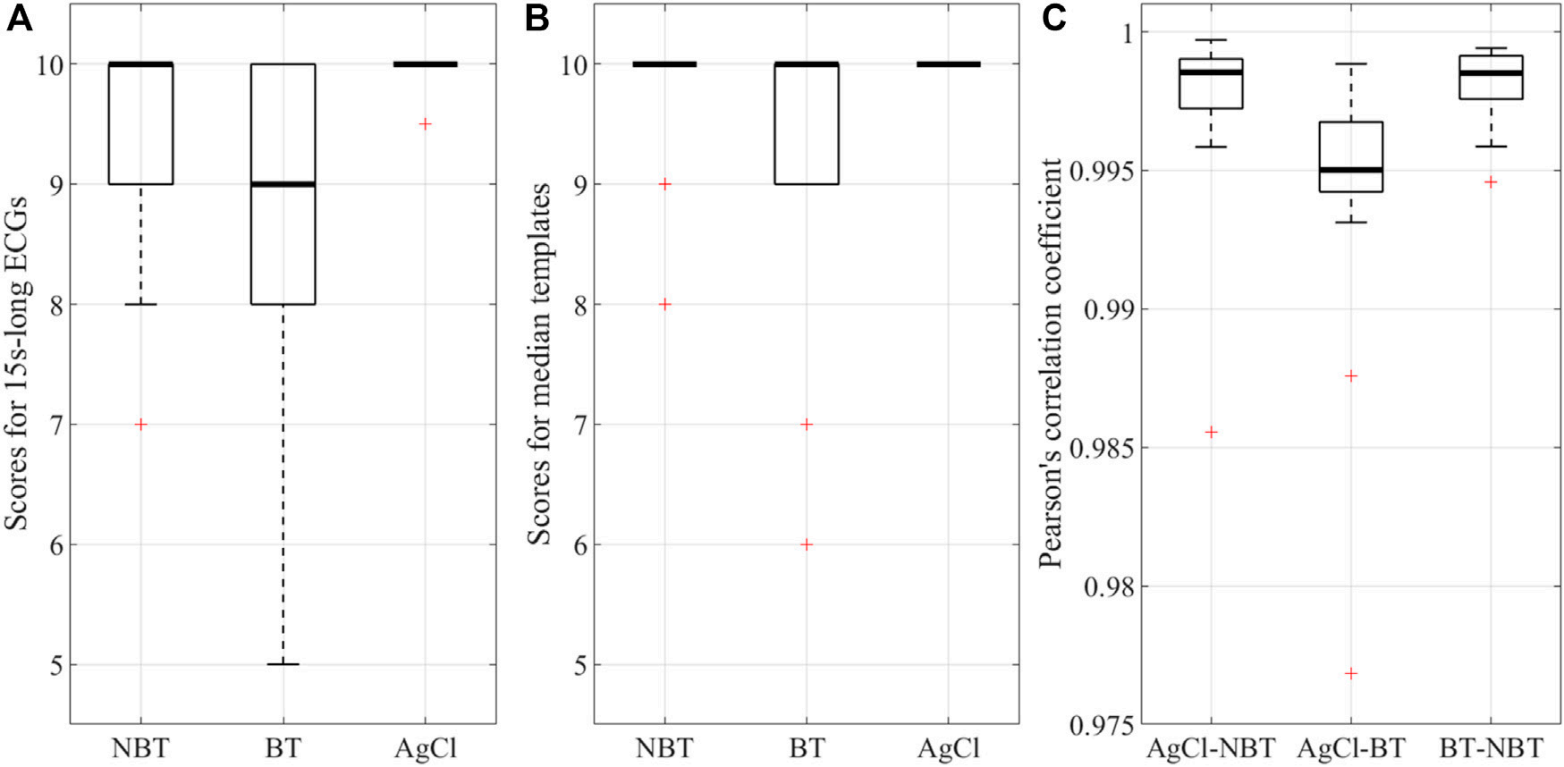
Qualitative scores (in the range 0–10) expressed for the **(A)** whole 15s-long ECG signals acquired by the three electrodes (NBT: non-breathable tattoo, BT: breathable tattoo, AgCl: gelled Ag/AgCl) and **(B)** their corresponding median beat templates. **(C)** The Person’s correlation coefficient computed between median beat templates acquired by the different electrode types: Ag/AgCl vs. non-breathable tattoo (AgCl-NBP), Ag/AgCl vs. breathable tattoo (AgCl-BT) and breathable vs. non-breathable tattoo (BT vs. NBT).

**FIGURE 8:**
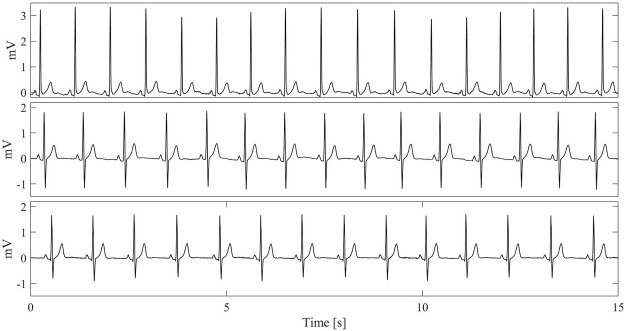
Examples of 15s-long ECG recordings with the highest qualitative score for the different electrode types (as such, they are taken from different subjects and not simultaneously). From top to bottom: lead II recorded by a pair of commercial gelled Ag/AgCl electrodes (upper plot), non-breathable tattoo electrodes (middle plot) and breathable tattoo electrodes (lower plot).

**FIGURE 9:**
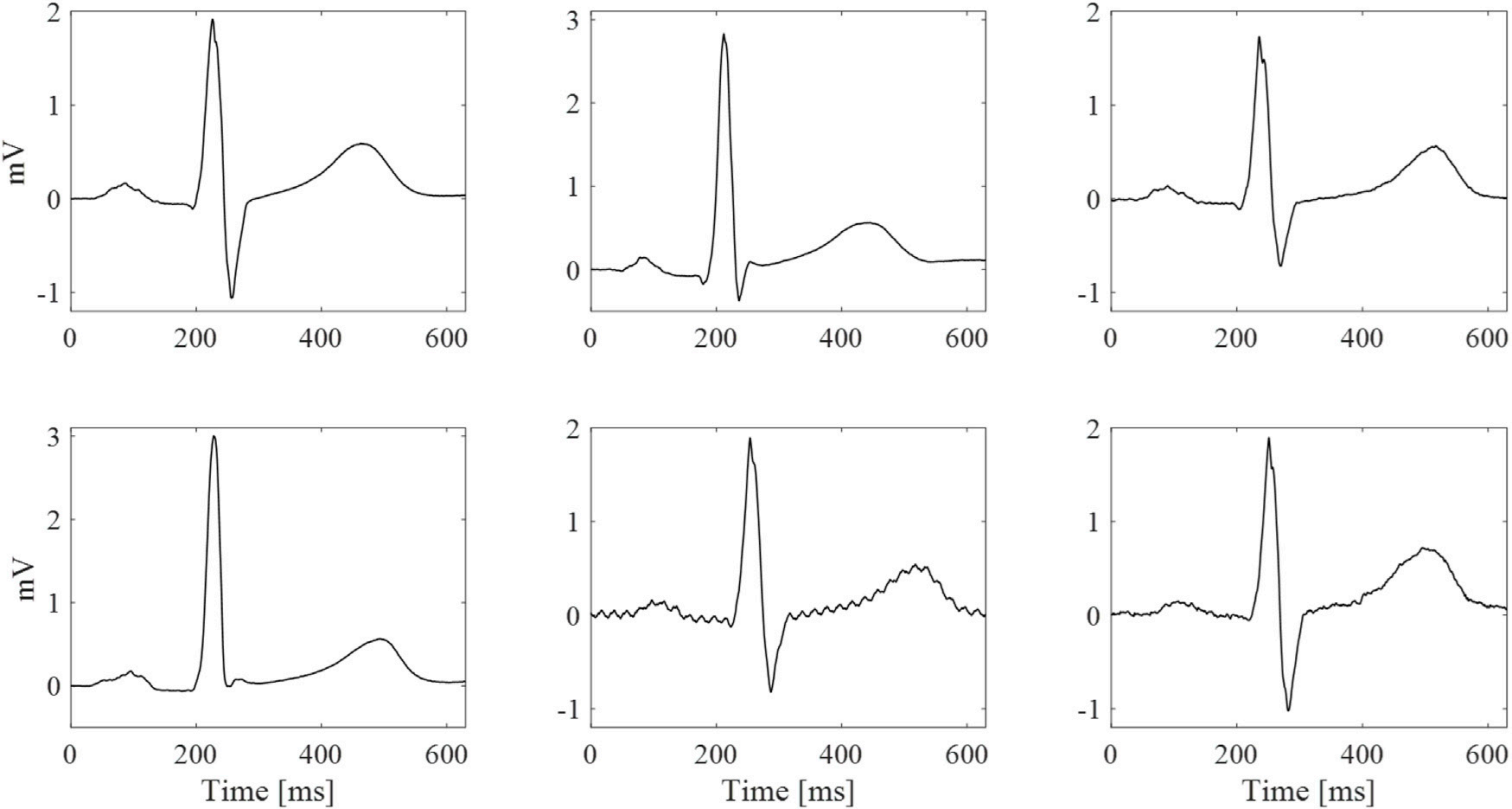
Examples of median beats with the highest (top row) and lowest (bottom row) qualitative scores for the different electrode types (taken from different subjects and not simultaneously). Left column: best commercial gelled Ag/AgCl electrode (score of 10/10 for both). Central column: non-breathable tattoo electrode (highest score 10/10 and lowest score 8/10). Right column: breathable tattoo electrode (highest score 10/10 and lowest score 6/10).

However, when looking at the high-frequency noise contributions and SNRs (Figure 6B, C), no statistical evidence was observed (*p* > 0.05), suggesting that tattoo electrodes, both breathable and non-breathable, show noisy contributions similar to Ag/AgCl electrodes. Remarkably, this outcome is achieved without removing the powerline interference and it is also confirmed by the qualitative scores provided by the clinician for the different high-pass filtered traces and their corresponding median beat template, which are reported in Figures 7A,B. The provided scores were generally greater than 8/10 for the 15 s-long signals and 9/10 for the median beats. Furthermore, the Pearson’s correlation coefficient computed between each pair of median beats was typically greater than 0.99, as shown in Figure 7C, thus suggesting remarkable similarity among the different templates, which is further confirmed in Figure 8 and Figure 9.

**FIGURE 10:**
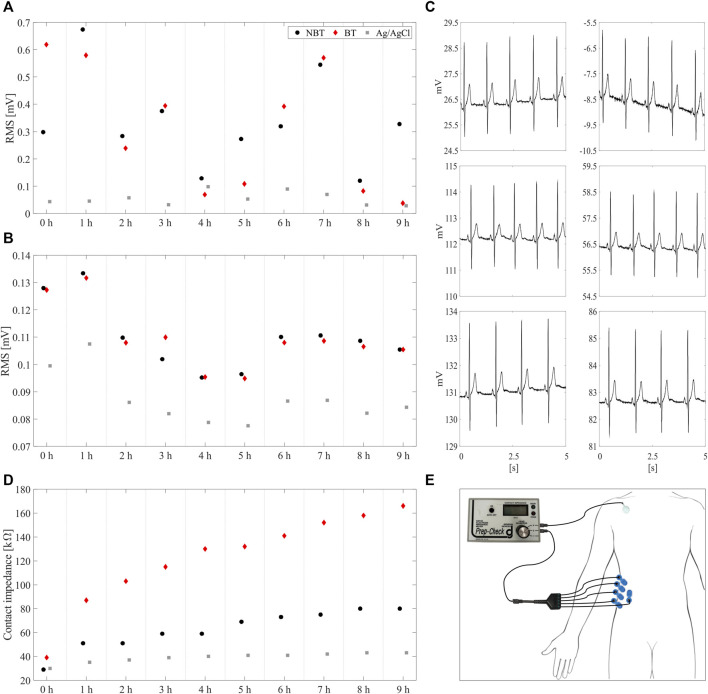
Root-mean-square level estimations for **(A)** low-frequency and **(B)** high-frequency noise performed on ECG signals recorded over 9 hours by the three different electrode types, i.e. non-breathable tattoo (NBT, •, breathable tattoo (BT, ♦) and gelled Ag/AgCl (■). In **(C)**, a 5 s-zoom on NBT (left column) and BT (right column) signals recorded at different hours, i.e. at 0 h (top), 4 h (middle) and 9 h (bottom), is reported. **(D)** evolution of the skin-electrode impedance. It can be noticed how the impedance relative to the skin-breathable electrode interface increases faster than the other two; this is due to the faster evaporation of the thin layer of sweat, which causes a faster drying out of the interface itself, thus a slight increase of the contact impedance. **(E)** Positioning of the electrodes for the impedance evaluation during the ECG acquisition.

A preliminary assessment of the performance of the electrodes during a 9-h span has also been performed in order to demonstrate the advantages offered by the proposed devices. Low-frequency and high-frequency noise estimations were reported in Figures 10A,B, respectively. The proposed electrodes behave quite homogeneously over time, as confirmed in Figure 10C, with a slightly worse performance compared to the adhesive electrodes with liquid electrolytic gel, as expected. During the whole experiment, the skin contact impedance has been also evaluated. As depicted in Figure 10D, the breathable electrodes showed a faster increase of the contact impedance with respect to both the non-breathable and the commercial one, due to the faster rate of sweat evaporation (Spanu et al., 2021). However, despite the faster degradation of the impedance, the breathable electrodes maintained excellent recording performances. The impedance was measured at 1-h intervals between the electrode under test and the parallel of five commercial pre-gelled electrodes (BlueSensor N, Ambu A/S, Denmark), as depicted in Figure 10E.

**FIGURE 11:**
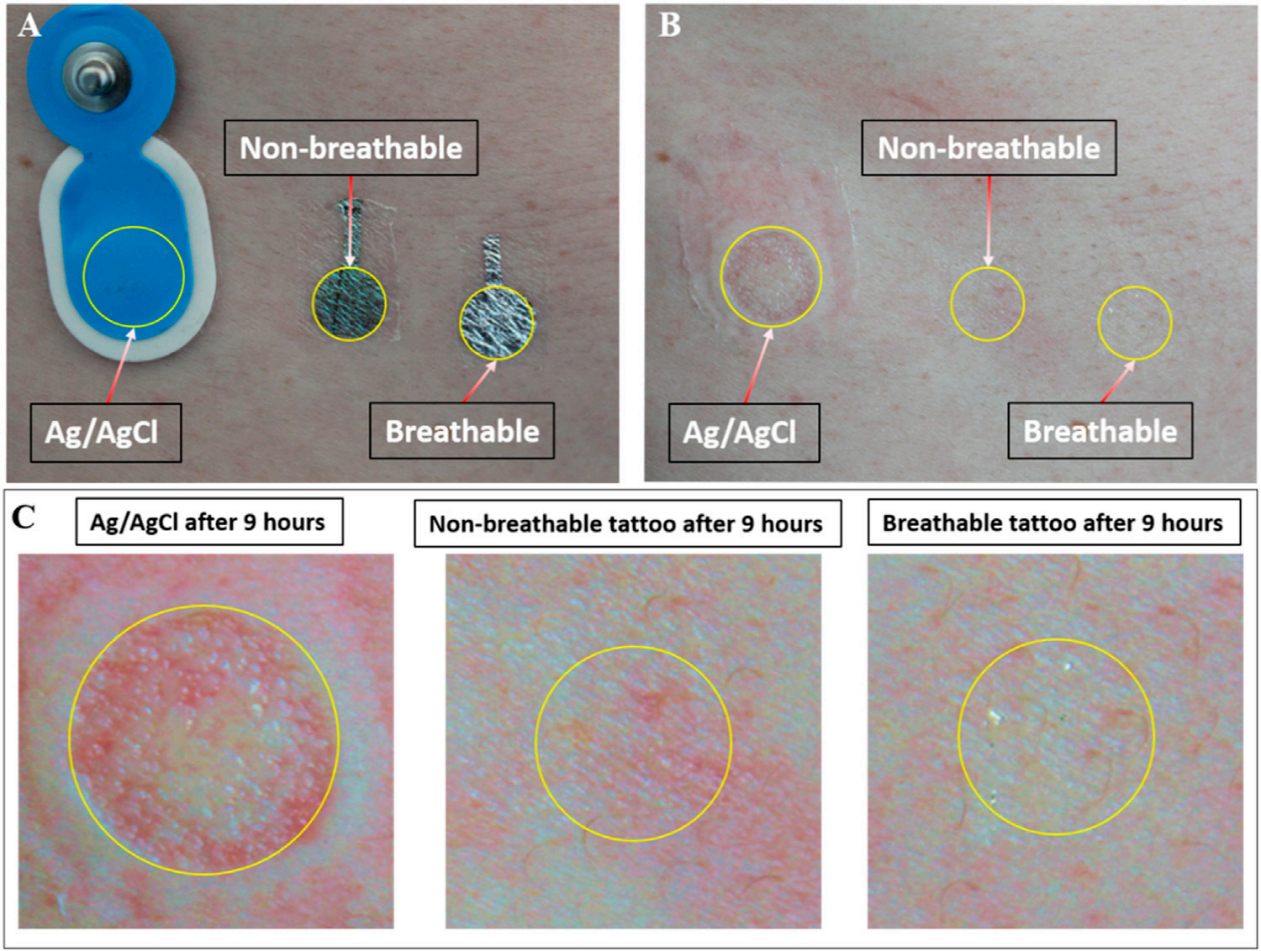
Effect of the electrodes on the skin. **(A)** The three types of electrodes placed on the right hip of the subject. **(B)** The same spot after the removal of the electrodes (9 h). **(C)** Magnification of the same area. Worse skin irritation was induced by the commercial electrode with respect to the Parylene C electrodes.

Interestingly, the tattoo electrodes showed a clearly better skin compatibility if compared with the commercial electrodes, as appreciated in Figure 11, where the effect of the three electrode types on light skin is presented. Even more interestingly, breathable tattoo electrodes showed almost no effect, even if compared with the non-breathable electrodes, thus confirming their potential for preserving the patient’s comfort during longer monitoring sessions.

Based on the previous analyses, the breathable and non-breathable tattoo electrodes offer good-quality ECG signals, affected by comparable noise contents with respect to commercial gelled Ag/AgCl electrodes, despite the statistical significance observed in low-frequency noise analysis. In this regard, the baseline wandering artefact is normally removed by linear and non-linear approaches (such as cubic-spline interpolation methods) in commercial ECG machines and, as such, its contribution is typically irrelevant in clinical recordings.

## Discussion

In this work, we demonstrate a novel, simple and upscalable fabrication technique of breathable and ultra-conformable dry tattoo electrodes based on perforated Parylene C nanofilms. Although different pore size and density may lead to different breathability levels, while also affecting the recording performance, such an investigation goes beyond the scope of this work. Conversely, these Parylene C electrodes present very good breathability, even overcoming that of common textiles and other materials used for realizing wearable sensors in contact with the skin, as well as excellent performances. In particular, in terms of recording quality, the analysis performed on both breathable and non-breathable tattoo electrodes, revealed comparable performance in terms of noise and SNR to commercial disposable gelled Ag/AgCl electrodes, as also confirmed by the cardiologist’s analysis, demonstrating the suitability of the Parylene C based electrodes for ECG detection and negligible impact of the perforation (at least at values of pore density and dimensions that ensure their breathability) on the quality of the detected electrical signal. Further analysis demonstrated that even after many hours, the quality of the bio-signal detected by the breathable electrode was not significantly worsened. Compared with both non-breathable electrodes made with the same materials and with commercial gelled electrodes, they showed a superior performance in terms of impact on the skin even after several hours. All of these elements corroborate the idea that this technology could represent a valid alternative to commercial electrodes in critical applications, such as bio-monitoring of elderly and new-born patients or intensive care of severely burnt people, where imperceptibility and breathability are of great importance. A critical aspect of this technology is the external connection with the recording devices because of the extremely low thickness of the electrodes. The proposed connection approach proved to be effective, allowing recording up to 9 h. However, further efforts are needed for the realization of better performing connections.

## Data Availability

The raw data supporting the conclusion of this article will be made available by the authors upon request, without undue reservation.
